# The Realization of Clinker-Reduced, Performance-Based Sustainable Concrete by the Micro-Filler, Eco-Filler Concept

**DOI:** 10.3390/ma14174958

**Published:** 2021-08-31

**Authors:** Joachim Juhart, Michael Autischer, Marlene Sakoparnig, Markus Krüger

**Affiliations:** Institute of Technology and Testing of Construction Materials, Graz University of Technology, 8010 Graz, Austria; michael.autischer@tugraz.at (M.A.); m.sakoparnig@tugraz.at (M.S.); krueger@tugraz.at (M.K.)

**Keywords:** sustainable concrete mix-design, performance-based design, durability, SCM, filler

## Abstract

In times of climate change, the reduction in embodied greenhouse gas emissions is a premise for sustainable concrete infrastructure. As Portland cement clinker is mainly responsible for the high CO_2_ emissions of concrete, its reduction is necessary. In order to be sustainable, the concrete must meet processing, mechanical and durability properties while taking cost aspects into account. The paper presents (i) the “micro-filler/eco-filler concept” for achieving a clinker reduced, optimised binder and (ii) a performance-based approach to put sustainable “Eco-concrete” into practice. Clinker is substituted by locally available inert fillers by at least two different particle size fractions and supplementary cementitious materials. The method is based on particle packing optimisation, reduction in water demand and optimisation of the mix ratio of the binder blend, which allows the performance requirements to be met. The new Eco-concretes deliver the desired performance in terms of processability, strength and durability (water penetration, frost, carbonation and chloride resistance) while lowering the environmental impact in comparison to standard concrete. One of the new mixes was used for a small animal passage tunnel. The direct comparison of the developed Eco-concrete and standard concrete showed a 24% reduction in CO_2_, while achieving satisfactory workability, stripping strength and durability performance.

## 1. Introduction

A maximum reduction in Portland Cement clinker in cementitious binders, cement and concrete is one major step on the pathway to the decarbonization of concrete production by 2050 [1]. This is due to the fact that clinker—which is the main constituent not just in ordinary Portland cement (OPC), but also in Portland composite cements, for example, according to EN 197-1 [2]—is primarily responsible for the global warming potential (GWP or CO_2_-eq.) and total primary energy demand (PEt) of normal concrete [3,4]. In contradiction to future technologies like CO_2_-capture and storage or clinker free, alternative cements, the development of binders and concrete with low clinker content is a practicable solution for decarbonization in building practice already today. It would help to achieve short-term CO_2_ reduction targets in 5 to 10 years. This requires either the provision of new, low-clinker composite cements or the mixing of existing cements with a high portion of supplementary cementitious materials (SCMs) and fillers to produce low-carbon concretes on an application-specific basis.

In the last few decades, the use of Portland composite cements (CEM II/A, CEM II/B) is increasing, e.g., in Germany and Austria [5,6]. The amount of clinker-substituting main constituents (i.e., SCMs) is limited to a maximum value of 35 wt.% in CEM II/B cements at the time [2]. However, extending the range to a maximum value of 50 wt.% of SCM in future CEM II/C is already foreseen in EN 197-5 [7]. Mixes of different SCMs such as ground-granulated blast-furnace slag (GGBFS), fly ash (FA), (micro)silica fume (MS), other pozzolans or tempered clays, etc., are possible. Additionally, slag cements (CEM III) with two main components, namely clinker and slag (S > 65 wt.% up to 95 wt.%) have been used in practice for many decades. However, the application of these cements in structural concrete is limited depending on the exposure class, in Austria according to ÖN EN 206 [8]. Additionally, the availability of slag as a clinker substitution is limited [9]. Such a restriction is also reported for fly ash use in some countries [9,10]. Therefore, the exploration of alternative SCMs and their appropriate use in blended binders is becoming increasingly important, especially those that improve durability properties such as chloride penetration resistance [11]. However, in the present paper, combinations of CEM I and different, actually available SCMs and fillers are presented in order to achieve a significant CO_2_-reduction in construction practice already today.

To ensure an adequate concrete performance, the pros and cons of different SCMs and fillers have to be considered. Latent hydraulically active and pozzolanic SCMs help to produce a dense microstructure during hydration and therefore will increase the resistance of concrete against chloride and chemical attack [12,13,14]. In addition, SCMs like GGBFS are by-products of industrial production and are considered to have a relatively low environmental impact [15]. On the other hand, mixes with a high amount of SCMs (esp. GGBFS) are known for slow (early) strength development and for a reduced carbonation resistance compared to pure OPC concretes [14]. The slow development of strength requires longer stripping times, which is not conducive to rapid work progress (e.g., stripping after 24 h). However, in order to call it sustainable, a clinker reduced concrete that is to be used in reinforced, infrastructural concrete construction should have the required, application-oriented and best feasible performance in terms of technical or functional aspects and ensure durability besides its low environmental impact. Performance-based design and life cycle assessment methods have to be combined to get the right basis for a multi-criteria decision [16,17]. According to Müller [18], the sustainability potential of building materials is influenced by (i) environmental impact, (ii) service lifetime and (iii) performance. As the desired lifetime of concrete structures ranges between 50 and 100 years (for infrastructure this can even be up to 200 years), it relativizes the environmental impact over a long time span. Consequently, high-durability building materials and structures increase sustainability. Attempting to reduce the environmental impact by clinker reduction without looking at the influence on the performance and durability of the material, could result in an increased environmental impact.

### 1.1. Micro-Filler/Eco-Filler Design Concept

To link workability, early strength (for stripping formwork), durability and environmental performance, a method, according to Juhart et al. [19], for designing eco-efficient binders combining OPC, selected secondary cementitious materials (SCMs) and inert fillers in an optimized way is used. The final mix design should be adequate for using it at ready-mixed concrete plants without significant machinery adjustments, except the requirement for available silo capacities for cement and 2 to 3 additives. An optimum of the mix proportions of all components and an optimum in terms of concrete properties is to be found, which is a multicriteria decision [16]. In particular, these criteria include an adequately good workability of the concrete, the desired strength for the earliest possible removal of formwork and for design load, appropriate resistance to environmental exposure, the lowest possible environmental impact and, last but not least, low (life cycle) costs.

**Figure 1:**
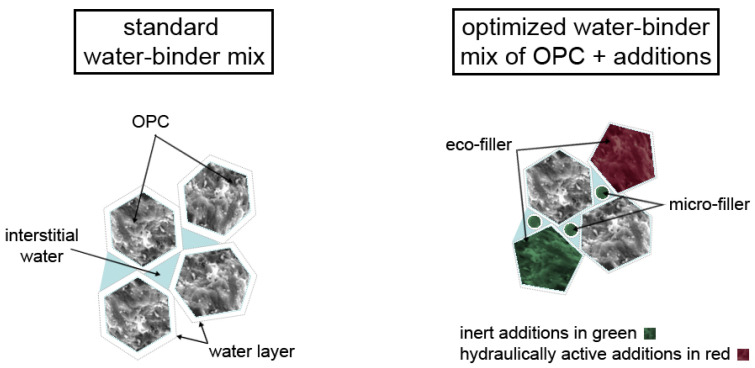
Optimizing paste by using combined fillers: normal paste of OPC and water versus eco-efficient paste with OPC, micro- and eco-fillers.

The performance-based mix design follows a stepwise approach: (i) suitable source materials selection, (ii) binder design with paste experiments by the micro-filler/eco-filler concept including tests to determine the compatibility of binders and additives and (iii) final development (upscaling) of the concrete mix as well as its performance proof in terms of workability, strength and durability. The general approach of optimizing binder composition is illustrated in Figure 1. The methodology is based on the optimization of packing density and minimization of water demand especially of the powders that form the paste (i.e., all granular materials with a maximum grain size < 125 µm) bearing in mind their environmental impact [19]. In an optimized paste OPC with its high GWP and PEt is partly substituted by properly selected very fine micro-fillers (MFs) and coarser eco-fillers (EF) that have low water demand and lower environmental impact. Both fillers can be hydraulically active or inert, so the physical filler effect as well as the potential latent-hydraulic or pozzolanic reactivity are to be considered in the mix design. Former studies [20,21] showed that a physical filler effect can be achieved by a size ratio of smaller particles to larger particles (i.e., MF to EF and OPC) d_MF_/d_EF/OPC_ ≤ 0.33 with an optimum reached at a ratio ⋍0.1. Practically MFs have a d_50_ < 3 µm in any case, significantly smaller than OPC. The physical filler effect increases the packing density of the mix and helps to accelerate hydraulic reactions inter alia by its nucleation effect and higher specific surface area (SSA) [22,23]. In this study, MFs are properly selected limestone (LS) or dolomite powders and EF are LS powders or GGBFS as pointed out in detail in Section 2.1. Eco-pastes typically have a decreased w/b_t_ ratio (water/total binder ratio) but the same or an even higher w/b_h_ ratio (water/hydraulically active binder ratio) compared to pure PC pastes. In general, their portion of clinker is reduced and thus their w/c ratio (water/clinker ratio) increases.

By means of the presented concept, optimal combinations of OPC and SCMs and finally—by applying the above steps (iii) and (iv)—optimal concrete mixes are found that are tailored to the requirements of the desired application.

### 1.2. Practical Implementation of the Design Concept

The concept presented was verified in practice by means of a prototype building, an animal passage tunnel under a railway line in Austria. All steps of realization were carried out, from concrete development, approval testing in a project-specific special admission, the dosage and way of adding of micro and eco-fillers in a ready-mix concrete plant and the delivery as well as concreting the prototype.

For direct comparison of the performance one half of the animal passage was manufactured with a well proven normal concrete as reference (Ref-C) and the other half with the Eco-concrete, as it will be described in the next sections. Both concretes should fulfil all requirements of concrete strength class C 25/30 and exposure classes XC4/XW1/XD2/XF3 according to ÖN B 4710-1 [24] (i.e., national specification of ÖN EN 206 [8]). This means, they shall provide soft and pumpable consistency (class F52) after a minimum processing time of 90 min, as they will be delivered as ready-mixed concrete by trucks. The exposure classes related to durability stand for high carbonation resistance (XC4), a maximum water penetration depth of 50 mm (XW1), high chloride resistance (XD2) and high resistance against frost attack without de-icing salts (XF3). A concrete structure’s design life of 100 years has to be considered.

Eco-concrete mix compositions can deviate from traditional concrete types and its prescribed requirements in the descriptive standards like minimum cement content or maximum water/cement ratio. Thus, they have to be designed and tested according to a performance-based approach. The scope of testing in such a performance-based design approach is much larger than the scope of testing of traditional standard concrete. In particular, the durability properties must be proven with suitable (accelerated) test methods and the interpretation of the test results has to be conducted with care, as the test methods were not developed and evaluated for such Eco-concretes in detail. For the building owner, implementation of performance-based, CO_2_-reduced concrete means a greater risk, since they must assume a concrete warranty for which little long-term experience is available, and which is outside of traditional standards. It is, therefore, particularly important to provide the owner with a comprehensive concept for testing and verifying the performance, as pointed out in the outlined paper.

## 2. Materials and Binder Design

Mixes were designed in steps: (i) characterizing and selecting appropriate powder materials (cements, SCMs, and fillers) considering fineness, clinker content or reactivity and ecological impact; (ii) designing eco-efficient binders or pastes by optimizing binder blends (combinations of OPC/EF/MF), water-binder ratios and SP-compatibility; and (iii) up-scaling the most promising blends to concrete and, finally, manufacturing them. In the last step, all project-specific relevant concrete properties and, in particular, the durability characteristics were tested and, if necessary, optimized by adapting the mix design.

### 2.1. Source Material Selection

The project-related standard normal concrete used as reference (Ref-C) contained a cement CEM II/A-L 42.5 N according to ÖN EN 197-1 [2] and a combination product “GGBFS-Mix” of GGBFS, limestone and fly ash as addition, which is commonly used in Austria according to Austrian Standard ÖN B 3309-1 [25]. For the two Eco-concrete variants considered in this study, cement CEM I 52.5 R, finely ground pure GGBFS and regionally available limestone powder as inert eco-filler (EF-LS with 97 wt.% CaCO_3_, 1 wt.% MgCO_3_) as well as 4 different micro-fillers were used. One of the micro-fillers is a dolomite powder (MF-DS with 54 wt.% CaCO_3_, 45 wt.% MgCO_3_) and the others are finely processed limestone powders of the same supplier. The latter products have a high content of CaCO_3_ (97–98 wt.%) and are essentially distinguished in their fineness according to Table 1. The product “MF-LS-D” has a plasticizing effect due to a special processing by the manufacturer, which enhances the flowability of binder blends if used.

To determine the clinker content of the binder blends, as discussed later, the minimum clinker content of 95 wt.% of CEM I and 80 wt.% of CEM II/A-L in ÖN EN 197-1 [2] was taken as the baseline. However, this clinker proportion is specified in relation to the amount of cement without the sulphate component. As the sulphate component is usually 4% of the total amount of cement, we subsequently specify the clinker content in relation to the total cement quantity as 91% (CEM I) and 77 wt.% (CEM II/A). The GGBFS mix contains roughly 80% of GGBFS, the rest is mainly LSP and a small amount of fly ash. Finally, the used CEM II/A-L contains a limestone powder content of about 15 wt.%.

**Figure 2:**
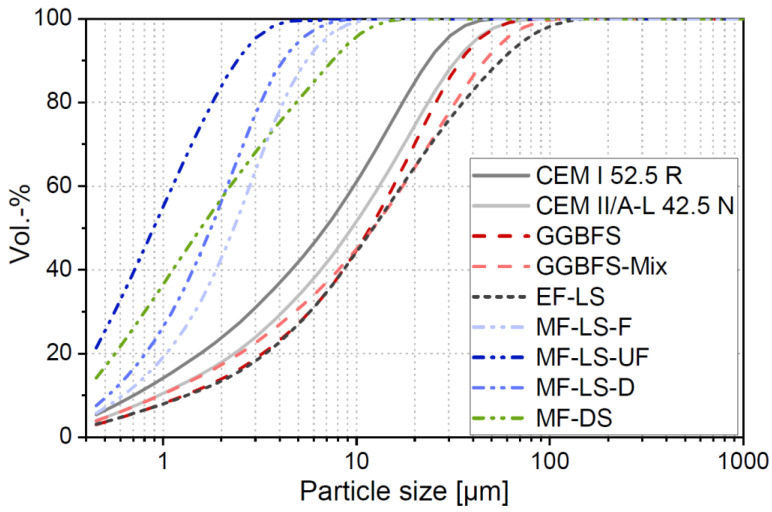
Particle-size distribution of the investigated binder materials.

A surfactant-based air entraining agent (AEA) and 4 different PCE-based superplasticizers depending on the binder and their compatibility were used. The powder materials were characterized including the determination of (i) dry particle density according to ÖN EN ISO 1183-1 [26], (ii) Blaine surface according to ÖN EN 196-6 [27], (iii) BET surface according of DIN ISO 9277 [28], (iv) particle-size distribution (PSD) carried out by a laser granulometric measurement (Sympatec Helos/Rodos with dry dispersion, result shows the mean value of 3 tests, with an measuring range between 0.45 and 875 µm), and (v) environmental indicators (PEt and GWP, see Section 2.2). The results are presented in Table 1 and Figure 2. The d_50_ value given in Table 1 is calculated from the PSD analysis and describes the particle size that 50 Vol.-% of the particles fall below. Characterization of the effectiveness and compatibility of SP´s to the different binder combinations was carried out by a compatibility check, which is explained in Section 2.3.2.

Carbonate aggregates (95–97 wt.% CaCO_3_, 1% MgCO_3_) in 3 fractions (rounded grain 0/4, 4/8 and 8/16) according to ÖNORM EN 12620 [29] were used in an optimized grading curve (close to the limit grading curve B of ÖNORM B 4710-1 [24]).

The PSD analysis in Figure 2 clearly shows that the EFs and the cements are in a typical size distribution range of ordinary cements. The four MFs show a greater variation of PSD and in general show greater fineness compared to the cements and EFs.

### 2.2. Ecological Impact of the Materials

**Figure 3:**
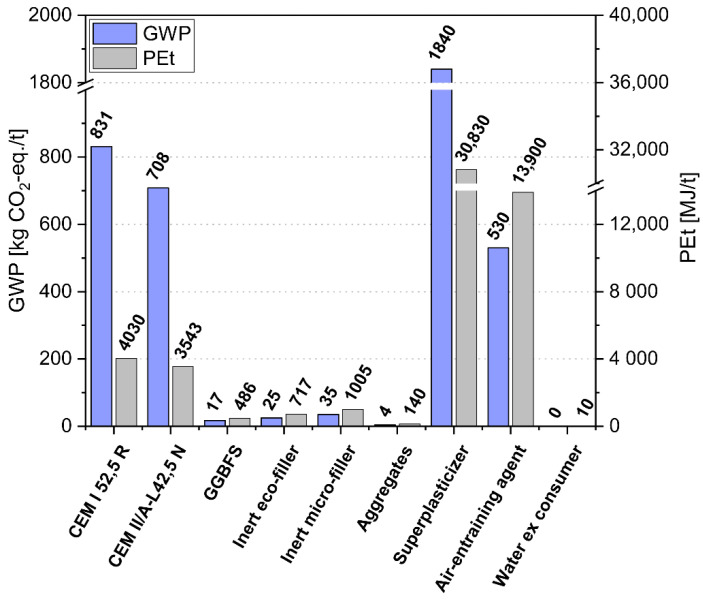
Environmental impact indicators of the various concrete constituents used.

Beside the physical requirements, the ecological impact is an important selection criterion in times of climate change with the main aim of reducing the CO_2_ emissions of concrete production. The considered data for the environmental indicators GWP and PEt of the used concrete constituents are shown in Figure 3. They were taken from equivalent materials published in [19] calculated by the method of Life Cycle Assessment (LCA) in accordance with ÖN EN ISO 14044 [30]. The production processes of constituent materials were modelled with input data from Ecoinvent 2.2 database using SimaPro software (v. 7.3). Evaluating in particular the milling and screening process of OPC and fine mineral powders showed that the energy consumption increases exponentially with decreasing fineness [19,31]. For modelling the different production impacts considering the powder fineness in a simplified way, two grades of average particle sizes were distinguished (d_50_ values of 2.8 µm for MF and 8.5 µm for EF, neglecting even greater fineness), resulting in GWP and PEt values of MF and EF according to Table 1 and Figure 3. However, cement far exceeds the environmental impact of finely milled stones (both types of fillers), aggregates and secondary raw materials (GGBS, FA), for which its clinker content is mainly responsible. SP and AEA possess very high GWP and PEt due to the energy demand of the production [32], which is clearly visible from the respective PEt values. The modelling of the source materials herein performed falls into the “cradle-to-gate” category, i.e., impacts associated with use and end-of-life, were not modelled. The ecological impact of 1 m^3^ fresh concrete was calculated in accordance with the values for GWP and PEt of the source materials and their fresh concrete quantities—the results can be found in Section 4.3. The environmental impacts of concrete production itself (due to operation of the mixing plant) and concrete transport were neglected, since these two shares of the total environmental impact are (i) relatively small (4% of GWP, 12% of PEt of an average Austrian concrete for example, [33]) and (ii) the same for all the mixes compared.

### 2.3. Binder Paste Design

Based on the combined filler or “micro-filler/eco-filler” concept [19], optimum mix ratios for the binder composition were worked out as follows. The concept aims to replace as much clinker as possible by additives with a lower CO_2_-eq., while achieving equivalent workability and strength to a standard binder as reference. In our case, the reference was the water–binder mix of the project´s usually desired normal concrete, i.e., Ref-paste with 88 wt.% CEM II/A-L 42.5 N and 12 wt.% GGBFS-mix. As a starting Eco-mix, a basic Eco-paste of 55% CEM I and 45% GGBFS was defined, which, from experience, is very workable and ecological, but develops strength more slowly than pure OPC [14]. To increase the packing density as well as the specific surface area (SSA) of the binder blend in order to accelerate strength development, GGBFS was systematically replaced by MF (3, 7 and 15 wt.% of binder). Additionally, the effect of a low-cost EF (15 wt.% of binder) was studied. The effectiveness of MF and EF dosage on (i) flowability and (ii) early strength was investigated on the one hand with blends at a constant w/b_t_ value of 0.50 and on the other hand at constant w/b_h_ value of 0.45 (Table 2). In the outlined study we define w/b_t_ as the water/total binder ratio (b_t_ is OPC, GGBFS, EF and MF), while, in contrast, w/b_h_ is the water/hydraulically active binder ratio, where all hydraulically active materials (b_h_ is OPC and GGBFS) are accounted for by 100% (i.e., k value of 1).

The cement paste variants (Table 2) were mixed in a 5-litre Hobart mixer with flat stirrer according to Hunger and Brouwers [34]. First, all the water and the entire amount of powder was added to the bowl. After 30 sec mixing at a low speed (140 ± 5 rpm), the mixer was stopped for 60 s to scratch splashed material from the wall of the bowl. To finish, the recipe was mixed again for 90 s at a low speed. The flowability of the pastes was tested by means of spread flow test according to ÖN EN 1015-3 [35] and Okamura [36] with a Haegermann cone on a dry glass plate without jolts (without compaction). The compressive strength was tested on prisms of hardened paste (40/40/160 mm) after demolding at an age of 24 h according to ÖN EN 196-1 [37]. Note that paste experiments were made without the addition of SP (for the SP selection, see Section 2.3.2).

#### 2.3.1. Derivation of Optimum Blends by Workability and Early Strength Assessment

**Figure 4:**
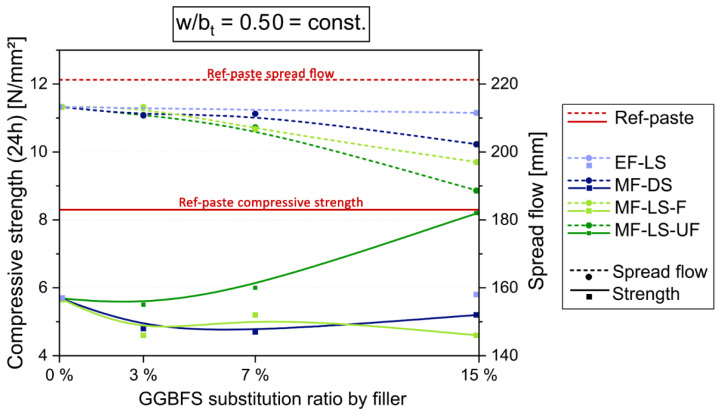
Compressive strength after 24 h and spread flow results from paste experiments with a const. w/b_t_ of 0.50. The content of CEM I is 55% in all mixes, GGBFS content varies by filler substitution (starting with 45% GGBFS at 0% filler).

The paste experiments at const. w/b_t_ of 0.50 in Figure 4 show that the spread flow decreases with increasing MF content and fineness (by substituting GGBFS to max. 15 wt.%). More than 15% MF would greatly reduce the flowability, especially of the finest MF-LS-UF. In contrast, EF does not influence or decrease spread flow significantly. Regarding early strength, the ultra-fine MF-LS-UF does increase it from a low substitution rate on (about 5%) more and more despite increasing w/b_h_. At 15% of MF-LS-UF the early strength value of Ref-paste can be reached (8.3 N/mm^2^). The coarser MF and EF do not have this potential, but keep the early strength from 3% to 15% substitution of GGBFS nearly at a constant level. The reasons for the different behavior are manifold and overlapping each other. Increasing packing density and specific surface area of the blend would increase strength, as they accelerate hydration. On the other hand, increasing w/b_h_ by greater GGBFS substitution-rates by inert LSP results in lower strength.

**Figure 5:**
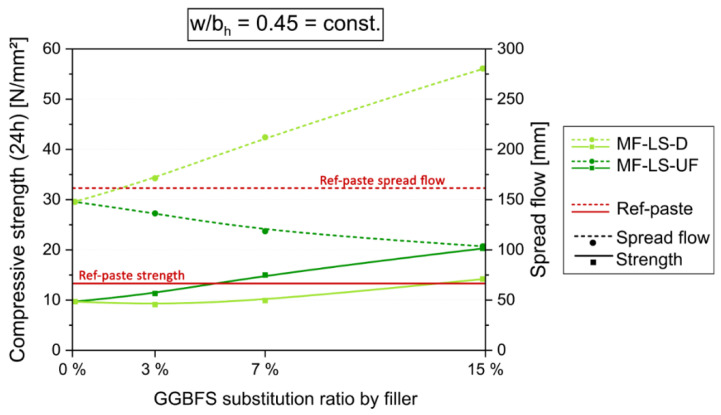
Compressive strength after 24 h and spread flow results from paste experiments with a const. w/b_h_ of 0.45. The content of CEM I is 55% in all mixes, GGBFS content varies by filler substitution (starting with 45% GGBFS at 0% filler).

For further optimization, the workability and early strength had to be adjusted with increasing MF content. For the first purpose, either suitable SP or an MF with plasticizing effect, i.e., MF-LS-D, could be used. For the latter purpose, w/b ratios can be reduced. Note that, in our case, no (fluid) SP was added in all the paste experiments, as this measure was reserved for further concrete development. Figure 5 shows the comparison of the effect of the stepwise substitution of GGBFS in the basic Eco-paste by MF-LS-UF and MF-LS-D at w/b_h_ = 0.45 = const. That means water content (and w/b_t_) is reduced with greater GGBFS substitution rates by inert LSP. For 15 wt.% of MF-LS-UF a doubling of the 24 h strength, but with negative influence on workability is achieved. However, an increased usage of MF-LS-D also results in increasing early strength while at the same time improving the workability. With an MF-LS-D content of 15%, an increase in early strength is achieved that is above the Ref-paste strength (14.2 N/mm^2^ > 13.3 N/mm^2^) while far exceeding its flowability (280 mm > 161 mm).

In the final evaluation of the results and based on the experiences of the former research [19,38], it was decided to further develop two Eco-paste variants with 5% resp. 15% substitution of GGBFS by LSP. One mix with the strength-increasing MF-LS-UF only and the other one with the economical EF-LS in combination with the plasticizing MF-LS-D.

#### 2.3.2. Superplasticizer-Compatibility

Compatibility tests according to ÖN EN 1015-3 [35] were conducted to evaluate the effect (liquefaction and consistency maintenance) of different SPs on the selected binder blends. While the standard SP and the SP dosage of Ref-C did not work satisfactorily with Eco-pastes, mixing two different SP-types properly resulted in highly effective liquefaction, good consistency keeping and similar viscosity, such as Ref-C of the Eco-pastes. According to the manufacturer, the first SP type has a strong liquefying effect, while the second has a consistency-keeping effect.

## 3. Concrete Mix-Design and Performance Testing

From the paste experiments the two most promising mixes were chosen to develop clinker-reduced, sustainable concrete: Firstly, the blend “UF Eco”, with 55 wt.% CEM I 52.5 R, 40 wt.% GGBFS and 5 wt.% of MF-LS-UF of the binder. It is characterized by a high content of hydraulic and latent-hydraulic binder (OPC + GGBFS) and—in order to increase packing density and early strength—a small amount of MF-LS-UF. Secondly, the blend “D Eco”, where the binder is composed of 55 wt.% CEM I 52.5 R, 30 wt.% GGBFS, 10 wt.% EF-LS and 5 wt.% of plasticizing MF-LS-D. The second approach would be very economical and would use GGBFS sparingly. GGBFS will become increasingly scarce in the future, when iron and steel production will produce less GGBFS because of new low-CO_2_ technologies [39].

The significant parameters of the mix composition are given in Table 3. According to ÖNORM B 4710-1 [24], a chargeable binder content b_c_ (cement is fully chargeable in it, GGBFS and GGBFS-mix with a k-factor of hydraulically activity of 0.8) of at least 300 kg/m^3^ and a water/binder (w/b_c_) value of maximum 0.53 is prescribed for the concrete type of the standard concrete C 25/30 XC4/XW1/XD2/XF3. Clinker was substituted by GGBFS and LSPs and water was reduced. Due to the reduced water dosage at constant total binder content (i.e., w/b_t_-reduction) of Eco-mixes, their paste volume decreased compared to the reference. The latter is defined as the volume of water, SP, air voids, binder and fines of the aggregates < 125 µm particle diameter. The w/b_t_ value of Eco-concretes was lowered in order to increase the early strength and to ensure sufficient durability potential, in particular, sufficient carbonation resistance despite clinker reduction. As a consequence, the w/b_h_ ratio remained constant (UF Eco) or increased slightly (+6% for D Eco) compared to the reference. For further comparison, the rough proportion of clinker and GGBFS in the total binder is given in Table 3 as well (see also Section 5). The AEA content and dosage of the pre-selected SP were adjusted to meet the requirements for air content and workability. The sieve lines of the aggregates of all mixes were the same. The grading curve was approximated to the recommended grading curve “B” in the favorable range according to ÖNORM B 4710-1 for a maximum grain size of 22 mm.

Two concrete mixtures of 1.5 m^3^ each of Ref-C, D-Eco and UF-Eco were mixed in a ready-mixed concrete plant in a double-shaft batch mixer DKX (BHS-Sonthofen) and transferred in quick succession to a truck mixer. For evaluation of the concrete properties, fresh concrete samples were taken from the truck mixer still on the site of the concrete manufacturer and tested. In addition, concrete specimens were produced and transferred into the laboratory for the strength and durability tests described below. In order to ensure that the two Eco-concretes demonstrably comply with requirements of the concrete C 25/30 F52 XC4/XW1/XD2/XF3 specified for the project, the following strength, workability and durability tests were conducted.

### 3.1. Workability, Air Content and Strength Test Methods

For consistency evaluation flow-table tests according to ÖN EN 12350-5 [40] were carried out directly after mixing, 10 min and 90 min after addition of water. The air content of fresh concrete was determined by the pressure equalization method according to ÖN EN 12350-7 [41] in parallel to the flow-table tests. Compressive strength tests were performed according to ÖN EN 12390-3 [42] on concrete cubes of 150 mm at an age of 1, 2, 7, 28 and 90 days. The results of 2 (at 1, 2, 7 and 90 days) or 3 (28 days) specimens were averaged. The 1 day-tests were carried out directly after demolding at an age of 24 h. The tests at 2 days were made after an additional 24 h storage under plastic foil in the lab at 20 °C. The other samples were stored under water until 7 days of age at 20 °C and then in standard lab conditions (20 °C and 65% r.h.).

### 3.2. Durability Test Methods

Durability checks include water penetration, freeze–thaw resistance, carbonation resistance and chloride diffusion.

#### 3.2.1. Water Penetration Depth

The water penetration was tested on three prisms (200/200/120 mm) according to ÖN EN 12390-8 [43] with specifications according to ONR 23303 [44]. The test specimens were roughened on the test surfaces with a steel brush before they were placed in underwater storage (up to the 28th day). After the underwater storage, the test surface, limited by a sealing ring (ø 10 cm), was exposed to a water pressure of 1.75 bar for 3 days and then to a water pressure of 7 bar for 11 days. After that, the samples were broken in half. Both water-penetrated partial areas of the surfaces were marked, and the mean penetration depth was evaluated.

#### 3.2.2. Freeze–Thaw Resistance

The freeze–thaw resistance was tested according to ONR 23303 [44] based on CEN TR 15177:2006 [45], “beam test” by sonic travel time measurements at concrete prisms (400 × 400 × 100 mm^3^). After demolding, the prisms were first stored in plastic sheets until the 7th day and from the 7th to the 28th day underwater. The sonic travel time before and after exposing 3 prisms of each mix to 56 freeze–thaw cycles was measured. Each cycle lasted 12 h and went through temperatures of +20 °C/−20 °C and back to +20 °C in a climatic chamber. The change of the sonic travel time after 56 cycles of each mix compared to that of a normatively produced “zero” concrete mix according to ONR 23303 [44] was evaluated.

#### 3.2.3. Carbonation

The carbonation resistance test was carried out according to ÖN EN 12390-12 [46], using the accelerated method. Two prisms (120/120/360 mm) were stored underwater until the 28th day and in lab-climate of 20 °C and 65% r.h. until the 42nd day. After such pre-storage to achieve medium moisture saturation, the samples were placed in a climate chamber at 20 °C, 57% r.h. and a CO_2_-content of 3%. To determine the carbonation depth at different exposure times of 0, 7, 28 and 70 days, slices were split off the prism (thickness of 7–8 mm) and phenolphthalein indicator solution was sprayed onto the fracture surfaces. The remaining pieces were each time returned to the climate chamber storage. 30–75 min after spraying, the carbonation depth was measured at 5 points at each of the 4 sides, with an accuracy of 0.5 mm. Outlier at pores and aggregates were not considered.

The natural carbonation rate was estimated according to ÖN EN 12390-12 [46] and as specified by Hunkeler [47,48], based on the measurement data from the accelerated carbonation process. That is to say a regression line was drawn through the measured carbonation depths d_k_ at different exposure time passing through the measured value at time t = 0 as fixed point. In Section 4.2.3 the square root of time is plotted on the *x*-axis, so that the linear slope of the regression line corresponds to the accelerated carbonation rate K_AC_ (in mm/√days). The conversion from the measured accelerated carbonation rate to a theoretical natural carbonation rate K_NAC_ is carried out by Equation (1) and the conversion parameters (Table 4) according to Hunkeler [47].

(1) $$K_{\mathrm{NAC}}=a\times b\times c\times K_{\mathrm{AC}}$$

#### 3.2.4. Chloride Penetration Resistance

The chloride penetration test was performed according to ÖN EN 12390-11 [49] using a 150 mm cube and evaluating the chloride diffusion coefficient. The cubes of each mixture were cut in half after storing them underwater. At one half powder-samples were taken from the cut area and the initial chloride content was determined. The other half was exposed to vacuum for 3 h and water-saturated in vacuum for a further hour. After that, all sides expect the cut surface were coated with a Cl^−^ free epoxy resin. After that, they were stored for 18 h in a saturated Ca(OH)_2_ solution before they were put into boxes filled with a 3% NaCl solution for 90 days. For the determination of the chloride diffusion coefficient, powder samples were obtained with a profile grinding machine by dry grinding of 9 individual layers starting from the exposed side to depth (0–25 mm). The chloride content of each layer was measured by potentiometric titration with silver nitrate according to ÖN EN 14629 [50]. The obtained Cl^−^ concentration values in various depths were used to calculate the diffusion coefficient (D_nss_) and the Cl^−^ surface concentration (C_S_) according to Equation (2) and Table 5 using a least-squares fitting method according to ÖN EN 12390-11 [49]. The obtained Cl^−^ profiles and fitted Cl^−^ diffusion curves are shown in Section 4.2.4. The value of the surface layer 0–1 mm has to be excluded for the fitting according to the standard.

(2) $$C_{X}=C_{i}\;+\;\left(C_{S}-C_{i}\right)\;\cdot \;\left(1-erf\left(\frac{x}{2\cdot \sqrt{D_{\mathrm{nss}}\cdot t}}\right)\right)$$

## 4. Results of Concrete Performance

Table 6 lists the results of Ref-C, UF Eco, D Eco and if applicable the normative limits for the parameters (i) workability (ii) strength (iii) water penetration depth (iv) sonic travel time (freeze–thaw resistance) (v) carbonation rate (vi) chloride penetration coefficient and (vii) ecological impact (GWP and PEt).

### 4.1. Workability, Air Content and Strength

**Figure 6:**
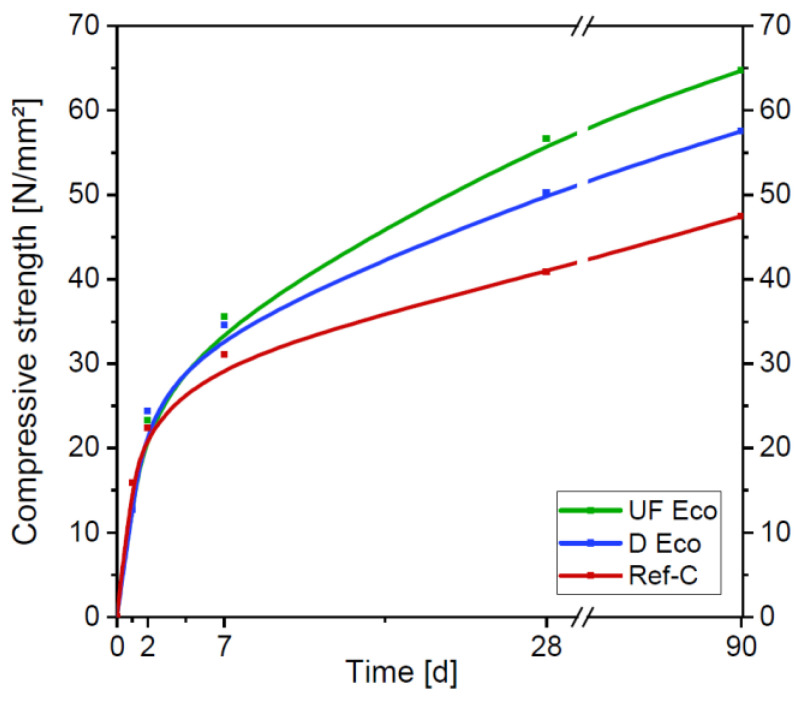
Compressive strength development of the Eco-concretes compared to Ref-C.

Consistency, air content and 28 d strength are the properties by which concrete is typically assigned to classes by limiting values (Table 6). They can be applied equally to traditional standard concrete and clinker-reduced “Eco-concrete”. According to ÖN B 4710-1 [24], the minimum flow-table spread for concrete class F52 (“soft consistency”) is 55 cm after 10 min and 49 cm after 90 min. All three concretes reached these desired values. Air content of Ref-C and UF Eco meets the specified range. The value of the D Eco concrete is above the limit, but was accepted due to its small excess. The compressive strength for concrete class C25/30 of ≥ 39 N/mm^2^ at an age of 28 d according to ÖN B 4710-1 [24] was reached (Ref-C) or even exceeded (Eco UF > Eco-D). Additionally, the construction company had set the goal of achieving the same early strength of standard concrete and reduced clinker concrete, in order to be able to progress with the construction work at the same rate. In Figure 6 the strength development of the concretes is compared. It can be seen that eco-concretes have a slightly lower early strength after one day, approximately the same early strength at an age of 2 d and, thereafter, higher strength than the standard normal concrete.

### 4.2. Durability Parameters

#### 4.2.1. Water Penetration Depth

The water penetration depth threshold of 50 mm (according to ÖN B 4710-1 [24]) is satisfied by all concretes by far. They had almost equal depths of water penetration (Table 6).

#### 4.2.2. Freeze–Thaw Resistance

The freeze–thaw resistance of all concretes is very close. The sonic travel time change of all variants is within the required range of differing not more than ±2.5% from the standardized zero concrete after 56 freeze–thaw cycles (according to ÖN B 4710-1 [24]).

#### 4.2.3. Carbonation

**Figure 7:**
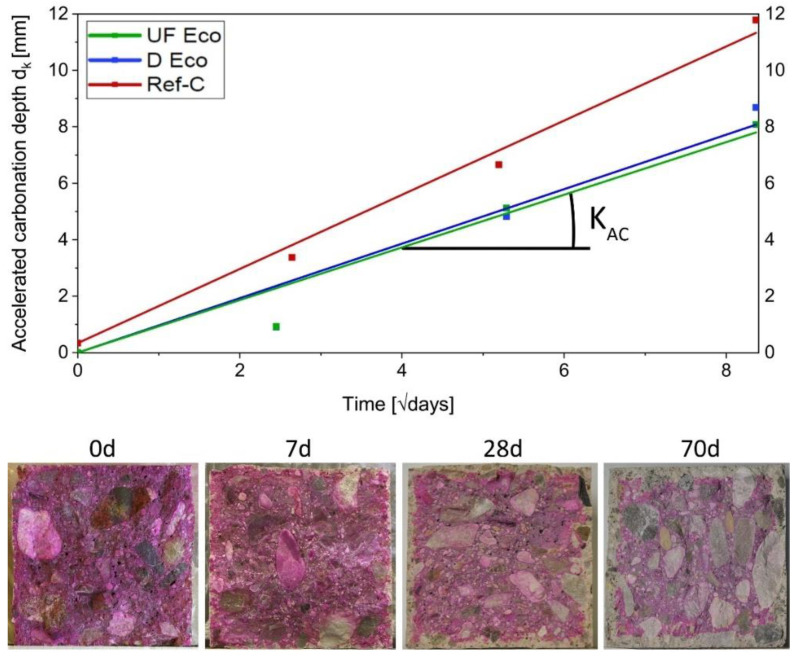
Accelerated carbonation rate of concrete variants and images of the test surfaces sprayed with phenolphthalein indicator solution at the corresponding test dates.

For structures with a life cycle of 100 years, the applied Swiss Standard [51] specifies a theoretical max. carbonation rate K_NAC_ of 4.5 mm/√year, which is undercut by all the concretes (Table 6). Both Eco-variants even show a better carbonation resistance than the standard concrete Ref-C (see Figure 7).

#### 4.2.4. Chloride Penetration Resistance

**Figure 8:**
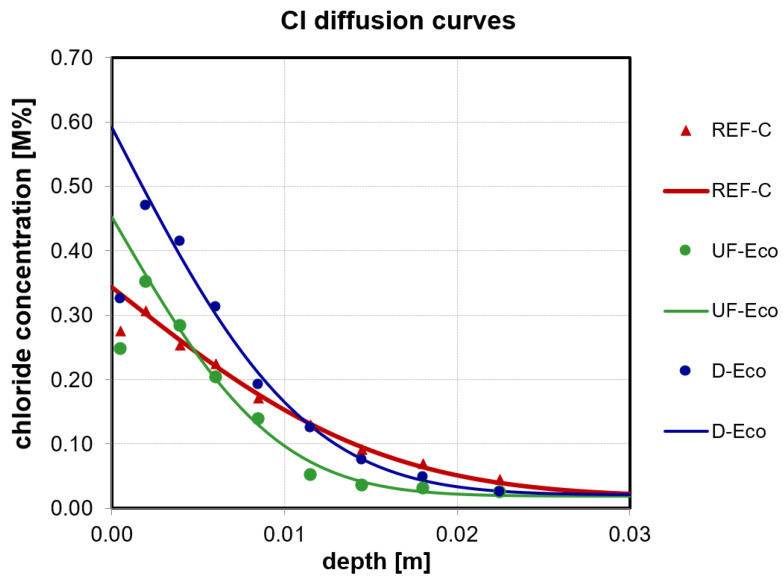
Measured chloride concentration with depth and chloride diffusion curves of the tested concrete mixtures. The chloride value from the first layer (0–1 mm) was not used for the fitting of the diffusion curve and the resulting diffusion coefficient.

As there is no limit value specified for the chloride diffusion coefficient as determined herein in the Austrian or another applicable European Standard, the results of the eco-concretes were referred to mixture Ref-C. The eco-concretes show slightly improved chloride resistance as their D_nss_ is lower (UF = 3.6 × 10^−12^ m^2^/s; D = 5.0 × 10^−12^ m^2^/s) than the D_nss_ of Ref-C (9.7 × 10^−12^ m^2^/s) (see Figure 8).

### 4.3. Ecological Impact

The “ecological impact” was calculated according to the values in Table 1 per m^3^ of fresh concrete. Due to the clinker reduction and the use of materials with a low CO_2_ rating, the GWP value could be reduced by 24% in both Eco variants. The PEt value could also be reduced by 17% and 18%, respectively, through the adjustments. SP had to be added a bit more to Eco-mixes than to the reference, which had a corresponding effect on the environmental impact of the eco-concretes due to its high GWP and PEt values, in total still very advantageous. With each m^3^ of concrete installed, more than 50 kg of CO_2_ could be saved, which corresponds to 2500 kg of CO_2_ for a quantity of 50 m^3^ installed.

## 5. Discussion and Practical Application

### 5.1. Binder Blends

The blended binders developed consist of 55 wt.% clinker and 45 wt.% of a mix of GGBFS and one or two LSP. Thus, they are equivalent to (future) CEM II/C cement composition according to EN 197-5 [7]. They have a much lower clinker content in the binder than currently used standard binders (i.e., CEM II/A plus additions of GGBFS, etc.) with approximately the same early strength and at least equivalent durability properties, as will be shown in Section 5.2. This means that the efficiency of the clinker and also the reactivity of GGBFS in the mix can be greatly increased by the MF/EF concept. In contrast to standardized cement, they are tailored to meet application-oriented requirements by the presented MF/EF concept. In this approach, very fine MF increase packing density, SSA and accelerate hydration due to a nucleation effect [22,23], while coarser EF increase ecological and economic efficiency. GGBFS as valuable, latent-hydraulically component from secondary resources helps to densify microstructure, improve durability and lowering environmental impact. The concept was successful applied to reach sufficient early strength equivalent to standard concrete for being able to strip formwork early (to reach daily or weekly cycles with removing formwork and building the next construction section).

### 5.2. Concrete Performance Evaluation

**Figure 9:**
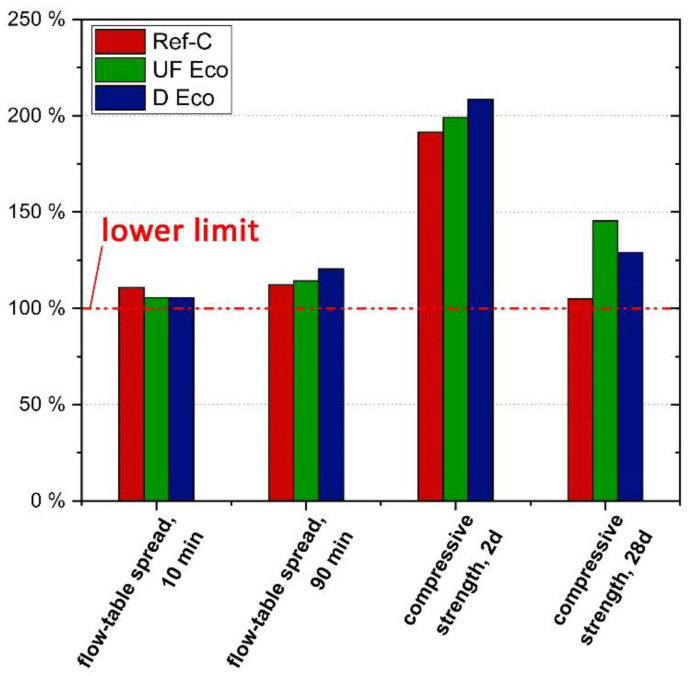
Performance indicators of standard concrete (Ref-C) and Eco-C variants related to standard requirements of workability and compressive strength (lower limit).

**Figure 10:**
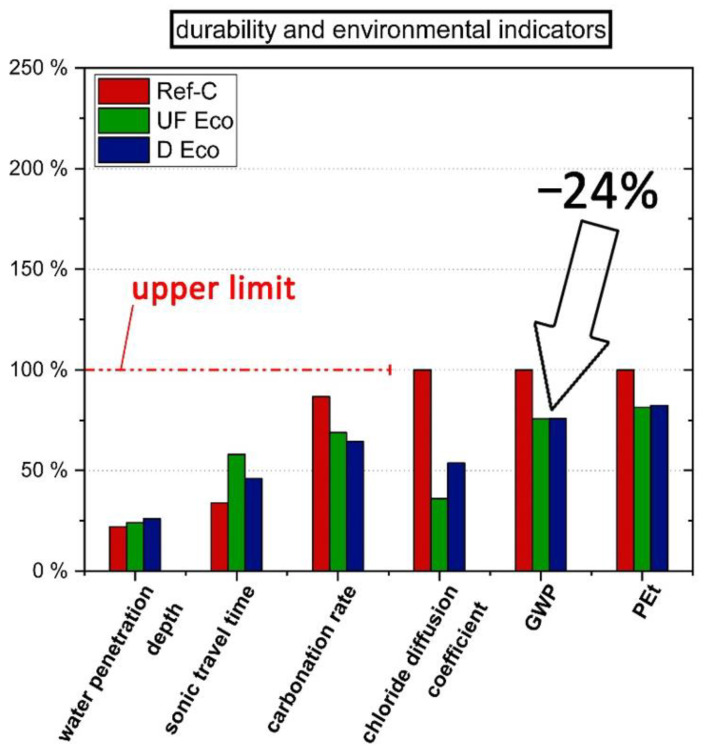
Performance indicators of standard concrete (Ref-C) and Eco-C variants related to standard requirements for durability and ecological performance of Eco-C compared to Ref-C.

The performance of the concretes was compared and evaluated in its entirety in order to be able to select the most suitable variant. For this purpose, the individual performance indicators (see Table 6) for the properties of workability (flow-table spread after 10 min and 90 min) and strength (2 d, 28 d-strength) as well as durability (water penetration, change in sonic travel time after 56 freeze–thaw cycles, carbonation rate and chloride diffusion coefficient) and environmental impact (GWP, PEt) were first normalized to the according requirements resp. reference concrete and then compared. If there are normative limits available for a specific property—these can be upper or lower limits depending on the performance characteristic—these limits were used as a reference value (=100%, see Figure 9 and Figure 10). Where no normative limits are specified, such as in relation to environmental impacts GWP and PEt, the value of the standard normal concrete was used as a reference to show whether the Eco-concretes are at least equivalent to it or better. One important criterion for the selection of concrete types is not mentioned here, namely the costs. Unfortunately, only incomplete and rough estimates of the production costs of the Eco-concretes are available from the manufacturer. While EF are generally cheaper than cement, the micro and ultra-fine micro-fillers may actually cost more than cement, especially as supply and demand are currently low. Furthermore, in the application case for Eco-concretes, an increased testing effort (durability tests) is currently needed compared to standard normal concrete. In terms of durability and life cycle costs, the existing Eco-concretes were designed to have at least the same durability as standard concrete (design service life of 100 years).

Figure 9 shows that the workability and strength of all concretes exceed the standard requirements as expected. It is remarkable that the consistency, i.e., the flow-table spread of the Eco-concretes exceeds that of Ref-C even after 90 min, despite their lower w/b_t_ value. The main reason for the good workability is the appropriate choice of type and dosage of SP. However, D Eco surpasses the limits after 90 min clearly, which means, SP dosage could be reduced a bit, especially regarding the “consistency keeping” part of the SP-mix.

It is also noteworthy that both Eco-concrete variants show a higher 2d strength than the standard normal concrete despite their low clinker content and high proportion of GGBFS. This is due to the above-mentioned effect of the fine or micro-fine limestone powders, which accelerate cement hydration. The goal of being able to strip formwork of eco-concretes at the same early stage as standard normal concrete was achieved. However, at 28 d and especially at the “high” age of 90 d, “overstrength” is achieved. In particular, the mix with the highest proportion of GGBFS (UF Eco) shows the highest strength (Figure 6). This means, that a GGBFS or even further clinker substitution by inert components (LSP) is possible and would lead to further reduction in embodied CO_2_ emissions. Moreover, if strength is related to clinker content, eco-concretes show a remarkably higher strength per wt. of clinker or higher clinker efficiency than standard concrete.

In terms of durability properties (Figure 10), all concretes are below the limits of the requirements given in standards in those cases where they exist. In terms of carbonation rate, Eco-concretes perform better than the standard normal concrete. This shows that even clinker-reduced concretes can have a very high carbonation resistance if the mix-design is suitable. There are no normative limit values for the chloride diffusion coefficient. However, it can be seen that the Eco-concretes with high content of GGBFS have a higher resistance than standard concrete. Such durability increases result in a prolonged lifetime, improving even more the LCA. Although such lifetime multiplier is not considered in the actual approach the aim of improving ecological performance is already achieved. GWP of Eco-concretes was reduced up to 24% and PEt up to 18% compared to Ref-C (Figure 10). It can be expected that further reductions in GWP and PEt are achievable if the performance of Eco-concrete is tailored even more precisely to the required limits in terms of functionality and durability.

In order to be able to design concrete with a minimum clinker content and correspondingly reduced CO_2_ emissions, the application of performance-based design is essential. The principle of “equivalent concrete (durability) performance” of Eco-concrete to standard concrete with traditional composition (in particular as specified in ÖN B 4710-1 [24] with prescribed reference cement type CEM II/A-L 42.5 N) hinders ecological improvement from being put into practice. Rather, the task of developing eco-efficient, sustainable concretes is a multi-criteria optimization and decision problem [16]. An optimum must be found in terms of functional-technical performance, durability, environmental impact and costs which is a multi-criteria decision. The building owner, client or public authority should decide how to weight the individual criteria and especially the environmental impact. For researchers and planners, it will be important to provide the appropriate basis for decision making. One possible approach was shown in the present project: take limit values if available or, if there are none, at least demonstrate equivalence with normal concrete, with a minimum environmental impact as the top optimization goal.

### 5.3. Practical Application in a Railway Infrastructure Project

**Figure 11:**
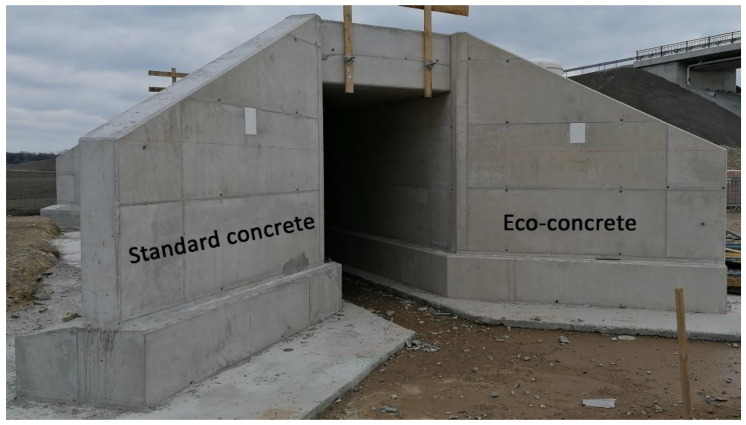
Entrance portal of the small animal subway. The left construction side in standard concrete and the right one in Eco-concrete (© Autischer, IMBT).

In order to test the performance of Eco-concrete under practical conditions, one of the developed Eco-concretes was applied in direct comparison to standard normal concrete. In the course of the double-track extension of a railway line, a subway for small animals with a clear cross-section of approx. 2.0 × 2.0 m^2^ and a length of about 15.0 m was built. About half of each construction part (foundations, walls, ceiling) were made of CO_2_-reduced concrete and the other half of standard concrete (Figure 11). Since both Eco-concrete variants were roughly equivalent in their overall functional-technical performance as discussed in Section 5.1, the production costs estimated by the concrete supplier were used as a basis for decision-making and the somewhat cheaper variant “D Eco” was chosen to be put in practice.

**Figure 12:**
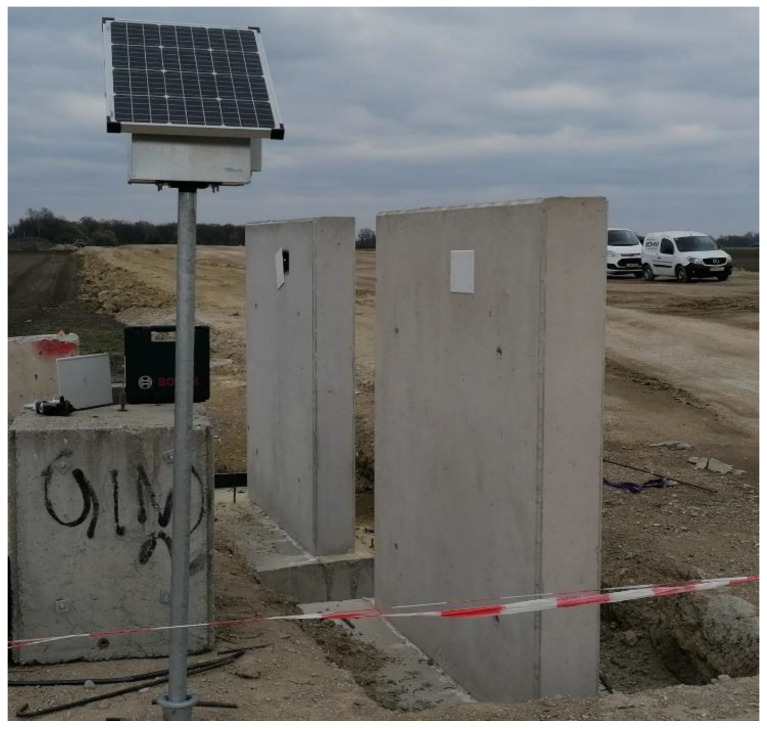
Mock-up walls in standard concrete and Eco-concrete for monitoring and long-term tests (© Autischer, IMBT).

In the course of execution, Eco concrete was produced in a conventional mixing plant for ready-mixed concrete and then delivered by truck mixers in the same way as standard concrete. Both concretes were placed shortly after each other (1–2 h) in the planned construction sections, which were foundations, walls and the ceiling. In addition, two mock-up-walls (see Figure 12) made of the two different concrete types were produced to allow for further investigations (sample taking and instrumented monitoring). Proper curing was applied for one week to prevent concrete from rapid desiccation.

With respect to workability, D Eco was equivalent to Ref-C. Only a somewhat faster stiffening at the execution temperatures of 27–30 °C on site compared to 20 °C in lab was observed. It was also noticed that Eco-concrete had a slightly higher viscosity than standard concrete. This corresponds to the lower water content and higher packing density of the fresh Eco-concrete compared to standard concrete.

**Figure 13:**
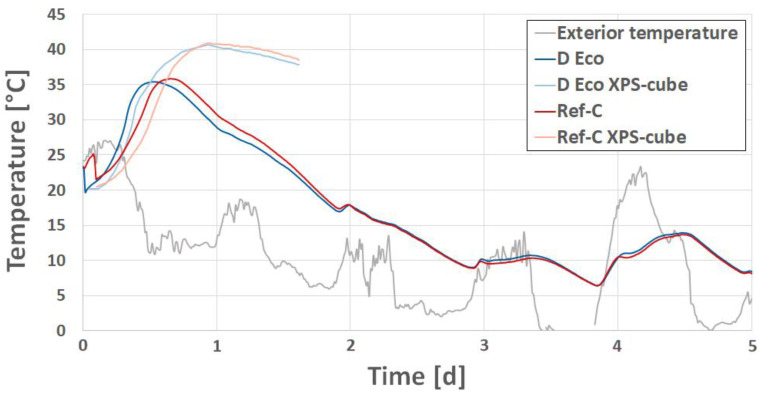
Hydration heat development within the mock-up walls and additional concrete cube specimens as well as environmental temperature during the first 5 days.

Hydration heat development was monitored by wireless sensors embedded in the mock-up walls as well as in some cube specimen that were used for strength evaluation. The results (see Figure 13) show no significant difference in heat development of the two concretes, which corresponds quite well to the expected strength development.

The desired fair-faced concrete quality was achieved for both variants, see Figure 11. In the course of the construction, it was proved, that Eco-concrete could be used equivalently to standard normal concrete in terms of practical construction issues (curing, etc.). Further investigations at the mock-ups in the next years will be used to evaluate the results shown here.

## 6. Conclusions and Outlook

In this study, Eco-concrete was designed by the MF/EF concept with the aim of optimizing its performance in respect to functionality, durability and environmental impact as the top optimization goal. Two Eco-concrete variants passed the performance tests with regard to the normative limits and proved to be at least equivalent to the standard normal concrete. At the same time the concept allowed a CO_2_ reduction of 24% compared to standard concrete. Further on, Eco-concrete was successfully applied in an infrastructure construction project. The practical implementation showed that the outlined, performance-based design concept for clinker-reduced concrete allowed to fulfil application-specific requirements as early formwork stripping and high durability. The production, processing, installation and stripping of the eco-concrete was to a large extent successful in the same way as that of the standard normal concrete. However, it was observed that the eco-concrete was somewhat more viscous and stiffened more quickly in the slightly warmer environment during execution compared to laboratory conditions.

We conclude that the principle of “equivalent concrete performance” of actual standards is not suitable for optimizing concrete in terms of CO_2_ reduction. Rather, limit values for performance requirements should be specified and met, while reducing the environmental impact and/or improving the service lifetime should be the top optimization goals. As concrete optimization is a multi-criteria decision, final selection of the concrete to be used is a question of weighting of individual criteria in an overall (owner’s) decision.

In a systematic monitoring of the structure, characteristic values of the damage progress (carbonation rate and corrosion potential) are to be recorded over several years and a model for service life prognosis will be derived from this.

## Figures and Tables

**Table 1:** Characteristic values of the investigated binder materials.

Material Category	Designation	d_50_	Dry Particle Density	Blaine ^1^	BET	GWP	PEt
		[µm]	[g/cm^3^]	[cm^2^/g]	[m^2^/g]	[kgCO_2_-eq./t]	[MJ/t]
Cement	CEM I 52.5 R	6.9	3.04	4897	1.8	831	4030
	CEM II/A-L 42.5 N	9.5	2.97	3977	1.5	708	3543
Eco-Filler (hydraulically effective)	GGBFS	11.8	2.83	3569	1.0	17	486
	GGBFS-Mix	12.2	2.71	4522	2.2		
Eco-filler (inert)	EF-LS	12.2	2.67	3480	1.6	25	717
Micro-Filler (MF)	MF-LS-D	1.8	2.61	(11,078)	2.6	35	1005
	MF-LS-F	2.3	2.61	-	-		
	MF-LS-UF	0.9	2.62	(19,498)	7.7		
	MF-DS	1.6	2.82	(12,773)	5.2		

^1^ The values in brackets are results outside the reliable measurement range of the Blaine method.

**Table 2:** Composition of the paste mixes.

Paste Mixes	CEM II	CEM I	GGBFS-Mix	GGBFS	Limestone-Powder	$\frac{w}{b_{t}}$/$\frac{w}{b_{h}}$
	[%]	[%]	[%]	[%]	[%]	[-]
Ref-paste	88	-	12	-	-	0.50/0.45
EF-LS	-	55	-	45/42/38/30	0/3/7/15	0.50/var.
MF-DS						0.50/var.
MF-LS-F						0.50/var.
MF-LS-D						var./0.45
MF-LS-UF						0.50/var.
MF-LS-UF						var./0.45

**Table 3:** Composition of the Ref-C and the two developed Eco-concrete mixes.

Material Designation	Unit	Ref-C	UF Eco	D Eco
CEM I 52.5 R	[kg/m^3^]	-	187	187
CEM II/A-L 42.5 N	[kg/m^3^]	300	-	-
GGBFS	[kg/m^3^]	-	136	102
GGBFS-Mix	[kg/m^3^]	40	-	-
EF-LS	[kg/m^3^]	-	-	34
MF-LS-D	[kg/m^3^]	-	-	17
MF-LS-UF	[kg/m^3^]	-	17	-
Gravel 0/4	[kg/m^3^]	986	984	995
Gravel 4/16	[kg/m^3^]	452	451	456
Gravel 16/32	[kg/m^3^]	452	451	456
SP (Ref-C)	[kg/m^3^]	1.87	-	-
SP (Eco-C)	[kg/m^3^]	-	2.38	2.38
AEA	[kg/m^3^]	0.68	0.41	0.51
Water	[kg/m^3^]	170	160	152
Fresh concrete density	[kg/m^3^]	2403	2389	2402
Total binder	[kg/m^3^]	340	340	340
Paste volume	[l/m^3^]	366	371	373
Clinker/total binder	[%]	68	50	50
w/b_h_	[-]	0.50	0.50	0.53
w/b_t_	[-]	0.50	0.47	0.45

**Table 4:** Conversion parameters (accelerated carbonation to natural carbonation according to Hunkeler [47]).

K_NAC_	Natural carbonation	
a	Conversion from 1 day to 1 year	19.1
b	Conversion from 3.0 to 0.04 vol.% CO_2,_ √0.04/3	0.12
c	Correction factor for accelerated carbonation (3% CO_2_)	1.30
K_AC_	Accelerated carbonation	

**Table 5:** Explanation of terms in Equation (2).

C_x_	Measured chloride concentration at depth x [wt.%]
C_i_	Initial chloride concentration of concrete [wt.%]
C_S_	Chloride surface concentration [wt.%]
x	Distance from the sample surface to the middle of the layer [m]
D_nss_	Non-steady state diffusion coefficient [m^2^/s]
t	time [s]

**Table 6:** Results and limits of the workability, strength, durability and ecological impact.

Investigation Parameters	Unit	Ref-C	UF Eco	D Eco	Limit Value
Flow-table spread 10 min	[cm]	61	58	58	≥55
Flow-table spread 90 min	[cm]	55	56	59	≥49
Air content 90 min	[%]	4.5	6.0	6.9	2.5–6.5
Compressive strength 1 d	[N/mm^2^]	15.9	12.9	12.7	Not defined
Compressive strength 2 d	[N/mm^2^]	22.4	23.3	24.4	>11.7
Compressive strength 7 d	[N/mm^2^]	31.1	35.6	34.6	Not defined
Compressive strength 28 d	[N/mm^2^]	40.9	56.7	50.3	≥39
Compressive strength 90 d	[N/mm^2^]	47.5	64.7	57.5	Not defined
Water penetration depth	[mm]	11	12	13	≤50
Sonic travel time	[%]	−0.8	0.4	−0.2	±2.5
Carbonation rate	[mm/√a]	3.8	2.8	2.7	≤4.5
Accelerated carbonation depth after 70d	[mm]	11.8	8.7	8.1	(≤13.2)
Chloride diffusion coefficient	[m/s^2^ × 10^−12^]	9.1	3.3	4.8	Not defined
GWP	[kg CO_2_/m^3^]	221	167	168	Not defined
PEt	[MJ/m^3^]	1261	1027	1037	Not defined

## Data Availability

Restrictions apply to the availability of these data. Data was obtained from Wopfinger Transportbeton GmbH and are available from the authors with the permission of Wopfinger Transportbeton GmbH.
